# Enantioselective Complexation of Xylopinine: A Cyclodextrin-Assisted CE and NMR Study

**DOI:** 10.3390/ijms26199405

**Published:** 2025-09-26

**Authors:** Erzsébet Várnagy, Gergő Tóth, Sándor Hosztafi, Milo Malanga, Ida Fejős, Szabolcs Béni

**Affiliations:** 1 Department of Pharmacognosy, Semmelweis University, Üllői út 26, H-1085 Budapest, Hungary; varnagy.erzsebet@phd.semmelweis.hu (E.V.); fejos.ida@semmelweis.hu (I.F.); 2 Center for Pharmacology and Drug Research & Development, Semmelweis University, Üllői út 26, H-1085 Budapest, Hungary; toth.gergo@semmelweis.hu (G.T.); hosztafi.sandor@semmelweis.hu (S.H.); 3 Department of Pharmaceutical Chemistry, Semmelweis University, Hőgyes Endre u. 9, H-1092 Budapest, Hungary; 4 CarboHyde Ltd., Berlini u. 47-49, H-1045 Budapest, Hungary; milo.malanga@carbohyde.com; 5 Integrative Health and Environmental Analysis Research Laboratory, Department of Analytical Chemistry, Institute of Chemistry, ELTE Eötvös Loránd University, Pázmány Péter Sétány 1/A, H-1117 Budapest, Hungary

**Keywords:** tetrahydroprotoberberine alkaloid, chiral separation, subetadex chiral selector, enantiomer migration order, ^1^H NMR titration, 2D ROESY NMR, diastereomeric splitting, inclusion complex, polar organic mode

## Abstract

Tetrahydroprotoberberine alkaloids (THPBs) are bioactive natural products bearing stereogenic centers that frequently exhibit enantiomer-specific pharmacological effects. Xylopinine (XPN), a representative THPB, shows cytotoxic, antimicrobial, and antimalarial activity in vitro, and displays pronounced stereoselectivity in vivo, with the naturally occurring (*S*)-enantiomer emphasizing the need for reliable enantioselective analysis. In this study, we present the synthesis of racemic XPN from norlaudanosine, and its first comprehensive cyclodextrin-assisted capillary electrophoresis screening dedicated to the enantioseparation of XPN. Sulfated- and sulfobutyl-ether-β-cyclodextrin (S-β-CyD, SBE-β-CyD) provided efficient resolution (*R_s_* > 3), while heptakis-(6-deoxy-6-(2-carboxyethyl)thio)-β-CyD (subetadex, SBX) yielded outstanding separation (*R_s_* > 9). The enantiomer migration order was consistently R,S, except when using SBE-β-CyD, which showed the inverse sequence. Chiral HPLC using a Chiralpak AD column in polar organic mode with methanol modified with 0.1% diethylamine as mobile phase enabled the semi-preparative isolation of XPN enantiomers, with the (S)-enantiomer exceeding 95% purity. The absolute configuration was confirmed by circular dichroism spectroscopy. ^1^H NMR titration and 2D rotating-frame nuclear Overhauser effect correlation spectroscopy (ROESY) consistently revealed multi-site recognition of XPN by SBX, supporting the inclusion of both aromatic rings (A and D).

## 1. Introduction

Protoberberine alkaloids (PBAs), including the extensively studied berberine, constitute a major subclass of isoquinoline-based natural products with diverse pharmacological activities, such as antibacterial, antioxidant, anticancer, and anti-inflammatory effects [1,2,3]. These compounds typically exist as quaternary ammonium salts and share a characteristic dibenzo [a, g] quinolizidine ring system biosynthetically derived from *L*-tyrosine via tetrahydroprotoberberine (THPB) intermediates such as (*S*)-scoulerine [4,5]. THPBs represent a structurally distinct group of PBAs that possess stereogenic centers and tertiary amine functionality.

**Figure 1:**
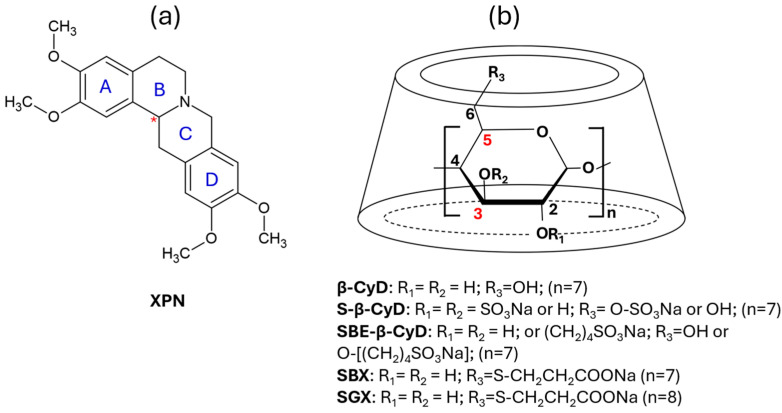
(**a**) Chemical structure of xylopinine (XPN) in its free base form, with the stereocenter indicated by an asterisk and annotated ring system (A–D). (**b**) Schematic representation of cyclodextrins (CyDs), highlighting the 3 and 5 positions on the glucose unit. For the abbreviation of the CyDs see Section 2.3 and Section 3.1.

Xylopinine (XPN), a naturally occurring THPB (see Figure 1), has been identified in various Annonaceae and Menispermaceae species, including *Xylopia laevigata* and *Stephania rotunda*. In vitro studies have demonstrated its cytotoxic, antimicrobial, and antimalarial properties, particularly against *Plasmodium falciparum* [6,7,8,9,10,11], the most lethal human parasite. Notably, its enantiomers exert opposing pharmacological effects in vivo: the (*S*)-enantiomer displays central depressant, anesthetic, and hypotensive activity, while the (*R*)-enantiomer induces sympathomimetic stimulation and pressor responses [12], which may be considered undesirable.

Such enantiomer-dependent behavior is consistent across several THPBs. For example, the (*S*)-enantiomer of tetrahydroberberrubine acts as a dopaminergic receptor antagonist, whereas its (*R*)-form is pharmacologically inactive [13,14,15,16]. These findings underscore the importance of developing robust enantioselective analytical methods for THPBs.

Chiral HPLC methods employing polysaccharide-type stationary phases have successfully resolved various THPBs, including tetrahydroberberine, tetrahydropalmatine [14,15,16,17,18]. In comparison, capillary electrophoresis (CE) has been rarely applied to this alkaloid class, despite offering several unique advantages: minimal sample and selector consumption, short analysis times, and compatibility with aqueous systems. Moreover, the modularity of CE allows rapid and cost-effective screening of diverse chiral selectors, particularly cyclodextrins (CyDs), via simple background electrolyte (BGE) modification.

CyDs act as host molecules capable of forming inclusion complexes that enhance the solubility, stability, and bioavailability of guest compounds [19,20]. Such a complex formation can be confirmed by NMR spectroscopy, particularly through rotating-frame nuclear Overhauser effect correlation spectroscopy (ROESY) correlations involving the inner-cavity H3 and H5 protons of the CyDs (see Figure 1). The properties of CyDs can be precisely tailored through chemical derivatization, including random or per-substitution. The latter includes single-isomer derivatives such as heptakis-(6-deoxy-6-(2-carboxyethyl)thio)-β-CyD (subetadex, SBX), featuring a uniform substitution pattern that greatly facilitates method reproducibility [21]. Due to their outstanding enantioselective recognition abilities, CyDs are among the most widely used chiral selectors in CE.

Although both partial and baseline resolution of tetrahydroberberine and tetrahydropalmatine enantiomers have been reported using 20 mM hydroxypropyl-β-cyclodextrin (HP-β-CyD), this remains the only CE method described in the literature [22,23], and no chiral CE methods have been reported for XPN. To address this gap, the present study aimed to develop a CyD-assisted CE method for the enantioseparation of XPN through systematic evaluation of CyD type and concentration on resolution and enantiomer migration order (EMO). Additionally, NMR spectroscopy was employed to characterize the binding interactions between XPN and selected CyDs.

## 2. Results and Discussion

### 2.1. Synthetic Procedures

**Scheme 1:**
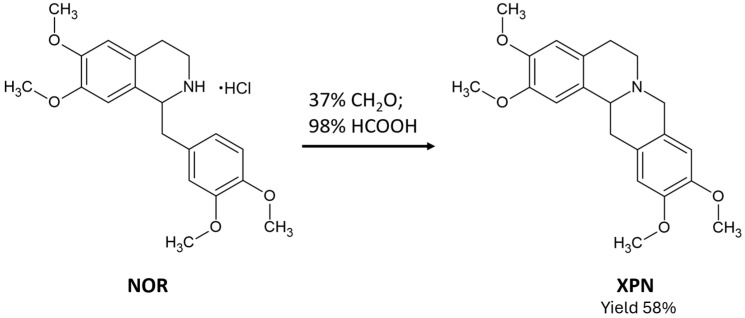
Streamlined synthesis of racemic XPN from NOR using Eschweiler–Clarke method, enabling direct access to the analyte for chiral separation studies.

Given its biological activity, XPN has been synthesized through various strategies reported in the literature [24,25,26,27]. To enable enantiomeric discrimination, both enantiomers of XPN were needed. Instead of pursuing a multi-step synthesis, we explored a simplified route starting from racemic norlaudanosine (1,2,3,4-tetrahydropapaverine, NOR), available from our previous work [28]. Heating NOR or its derivative with formaldehyde in the presence of formic acid (Eschweiler–Clarke conditions) induced ring closure [26,29,30] and, in our case, afforded racemic XPN in good yield in a single step (Scheme 1). For detailed description of the synthesis, see Section 3.2. The obtained racemic product served as the analyte for chiral separation studies.

### 2.2. Semi-Preparative Isolation of XPN Enantiomers

Based on previous reports demonstrating the feasibility of chiral HPLC for THPBs [18,19], we aimed to establish a practical semi-preparative method for isolating XPN enantiomers in high purity. To this end, we first screened the enantioselectivity of six analytical chiral stationary phases in polar organic mode, which offers the practical advantage of easy solvent removal during subsequent processing [31]. Among the tested columns, three stationary phases were CyD-based: Shiseido Chiral CyD-pH (phenylcarbamate-β-CyD), Astec Cyclobond I 2000 (native β-CyD), and Nucleodex β-PM (permethylated β-CyD). The other three were polysaccharide-based: Chiralpak AD and Chiralpak IA (both containing amylose tris(3,5-dimethylphenylcarbamate), with Chiralpak AD using the coated form and Chiralpak IA featuring the immobilized derivative), and Chiralcel OD (cellulose tris(3,5-dimethylphenylcarbamate)). The mobile phases consisted of acetonitrile (ACN) and methanol (MeOH), both modified with 0.1% diethylamine (DEA) to account for the basic nature of XPN. Overall, the polysaccharide-based columns showed superior enantiorecognition compared to the CyD-based columns. While no enantioseparation was observed on native or permethylated β-CyD columns, the phenyl carbamate-β-CyD based stationary phase provided above-baseline separation (see Table 1). The maximum resolution was obtained using the Chiralpak AD column with a MeOH-based mobile phase, consistent with our previous results observed for the structurally related benzyl-tetrahydroisoquinoline alkaloid, NOR [28].

**Figure 2:**
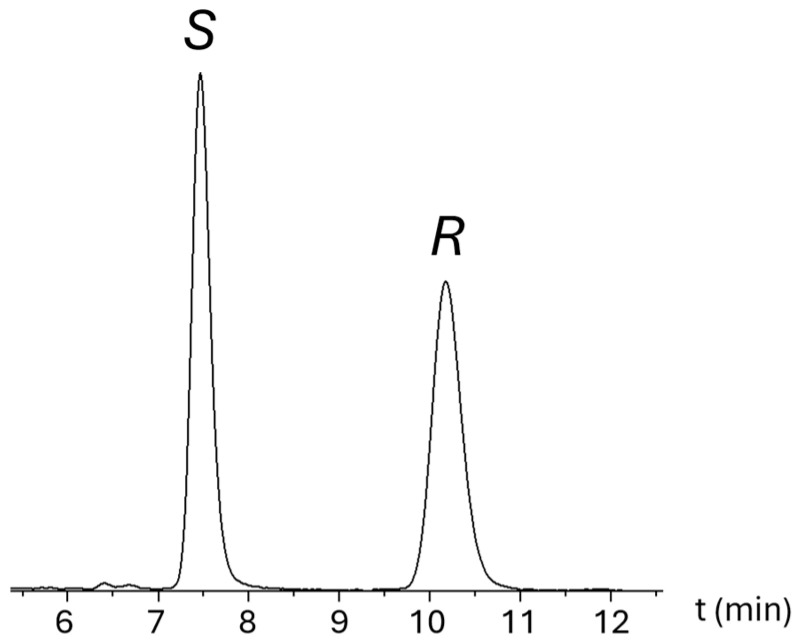
HPLC chromatogram representing the enantiomer elution order applying Chiralpak AD column, MeOH mobile phase modified with 0.1% diethylamine. Further conditions can be found in Section 3.3.

Given its superior performance, the Chiralpak AD column was selected for semi-preparative enantioseparation of XPN. Following isolation, the absolute configuration of the enantiomers was determined using circular dichroism spectroscopy, with (*S*)-NOR employed as a reference (see Supplementary Materials Figures S1 and S2). The first eluting enantiomer on the Chiralpak AD column was identified as (*S*)-XPN, followed by (*R*)-XPN (see Figure 2). The enantiomeric purity of isolated (*S*)-XPN exceeded 95%, while the (*R*)-enantiomer exhibited slightly lower purity.

### 2.3. CyD-Mediated Capillary Electrophoretic Study of XPN

Chiral separation of THPBs by CE presents significant challenges. For instance, tetrahydroberberine achieved only partial resolution (*R_s_* 1.2) using 20 mM HP-β-CyD at pH 2.5 [22]. Notably, the same selector and concentration were applied in a CE–MS method for the enantioselective quantification of tetrahydroberberine and tetrahydropalmatine in botanical extracts, demonstrating the potential utility of this technique in phytochemical analysis [23].

In contrast, structurally related benzyl-tetrahydroisoquinoline alkaloids, such as LAU and its derivatives, have been successfully resolved by CE using various CyDs, with resolution values (*R_s_*) exceeding 1.5. Among these, sulfated γ-CyDs demonstrated outstanding performance, reaching *R_s_* values above 10. Notably, chiral selector such as octakis-(6-deoxy-6-(2-carboxyethyl)thio)-γ-CyD (sugammadex, SGX) provided consistently high resolution for all four tested LAU analogs [28].

Guided by these findings, initial CE experiments with XPN were performed under similar conditions (pH 7.4), where XPN remains cationic, enabling applicability in CE and the use of neutral CyDs.

A systematic screening of over 20 native and derivatized CyDs was conducted to investigate the enantiomeric discrimination ability toward XPN. Among native CyDs, α- and γ-CyDs were ineffective, while β-CyD provided near-baseline resolution (*R_s_* 1.4). Compared to the modest selectivity observed for tetrahydroberberine, HP-β-CyD failed to separate the enantiomers of XPN, suggesting that specific structural features of XPN may hinder effective complexation with hydroxypropyl-substituted CyDs.

Several randomly substituted (e.g., sulfated-β-CyD (S-β-CyD), sulfobutylether-β-CyD (SBE-β-CyD), carboxymethyl-β-CyD (CM-β-CyD)) and single-isomer sulfated β-CyDs (e.g., heptakis-(6-*O*-sulfo)-β-CyD (HS-β-CyD), heptakis-(2,3-*O*-dimethyl-6-*O*-sulfo)-β-CyD (HDMS), heptakis-(2,3-*O*-diacetyl-6-*O*-sulfo)-β-CyD (HDAS), heptakis-(2-*O*-methyl-3,6-*O*-disulfo)-β-CyD (HMDiSu), hexakis-(2,3-*O*-dimethyl-6-*O*-sulfo)-α-CyD (HxDMS), octakis-(2,3-*O*-dimethyl-6-*O*-sulfo)γ-CyD (ODMS)) resulted in only modest enantiomeric separation (*R_s_* < 1). This was likely due to the close migration of the XPN–CyD complexes to the electroosmotic flow (EOF) at pH 7.4, which resulted in peak distortion. Therefore, additional measurements were conducted at lower pH using only the most promising CyD candidates. At pH 4.5 (20 mM acetate–TRIS buffer), S-β-CyD and SBE-β-CyD provided significantly improved resolutions of 3.2 and 4.6, respectively.

Sugammadex analog CyDs cannot be measured at acidic pH due to their limited water solubility, which is optimal between pH 7 and 8. In the case of SGX, we observed near baseline resolution at pH 7.4. The highest resolution (*R_s_* > 9) was achieved using SBX at pH 7.4 (see Table 2). Compared to native β-CyD, the introduction of 2-carboxyethylthio side chains in SBX markedly enhanced the enantioselectivity.

To gain insights into the separation mechanism, apparent averaged complex stability constants (*K_stab_*) and complex mobilities were also determined for the β-CyD and SBX complexes (see Supplementary Materials, Table S1). For β-CyD, moderate-stability constants were obtained for both enantiomers (*K_stab_* 280–380 M^−1^), indicating relatively weak and comparable binding affinities. Selected electropherograms from the β-CyD titration of XPN, recorded at various β-CyD concentrations, are shown in Figure S3.

In contrast, the SBX selector exhibited markedly stronger host–guest interactions, with stability constants exceeding 2100 M^−1^ for (*R*)-XPN and 3800 M^−1^ for (*S*)-XPN (Table S1). The selected electropherograms, recorded at various SBX concentrations, are presented in Figure S4. The pronounced difference in complex stability accounts for the superior resolution achieved in CE, as the separation was affinity-driven (disparity in binding affinities).

**Figure 3:**
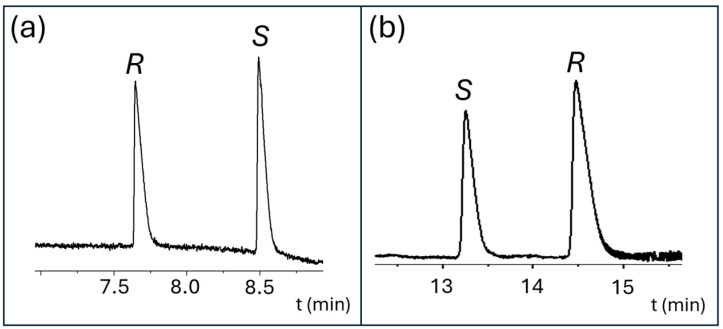
Demonstrative electropherograms depicting the substituent-dependent EMO reversal for XPN comparing (**a**) SBX and (**b**) SBE-β-CyD. Further conditions can be found in Section 3.5. For the abbreviation of the CyDs, see Section 2.3 and Section 3.1.

Migration order analysis revealed a consistent *R*,*S* order for all CyD selectors tested, except for SBE-β-CyD, which exhibited an inverse sequence (see Figure 3), due to differences in CyD substitution. Since the (*S*)-enantiomer represents the naturally occurring form in plant sources, most of these CyDs—excluding SBE-β-CyD—are suitable for reliable enantiomeric excess determinations in both pharmaceutical and phytochemical contexts. This EMO ensures that the minor peak (the impurity) migrates first, avoiding overlap with the main peak and enabling accurate quantification in the case of enantiomeric excess [32].

### 2.4. Characterization of the CyD Complexes by NMR Spectroscopy

The formation of CyD inclusion complexes with PBAs has been demonstrated using several techniques [33,34]. For example, complexation of oxyberberine with HP-β-CyD has been confirmed by infrared spectroscopy, differential scanning calorimetry, X-ray, NMR, and molecular modeling, while interactions between SBE-β-CyD and berberine analogs have been supported by isothermal titration calorimetry and fluorescence spectroscopy [35,36]. For benzyl-tetrahydroisoquinoline-type alkaloids such as LAU, NMR studies revealed that the aromatic C-ring (corresponding to the D-ring in XPN) can be accommodated in the cavity of SGX [28].

Following the successful enantioseparation of XPN in CE, we performed further characterization by ^1^H NMR titration and ROESY experiments. Due to the limited aqueous solubility of XPN, all NMR measurements were conducted in pD 6.0 buffer. Complete ^1^H signal assignment of racemic XPN was carried out under these conditions. Selective 1D ROESY experiments were used to support unambiguous signal assignment (see Supplementary Materials Table S2 and Figures S5–S9). The resulting chemical shift values served as references for monitoring host–guest interactions.

**Figure 4:**
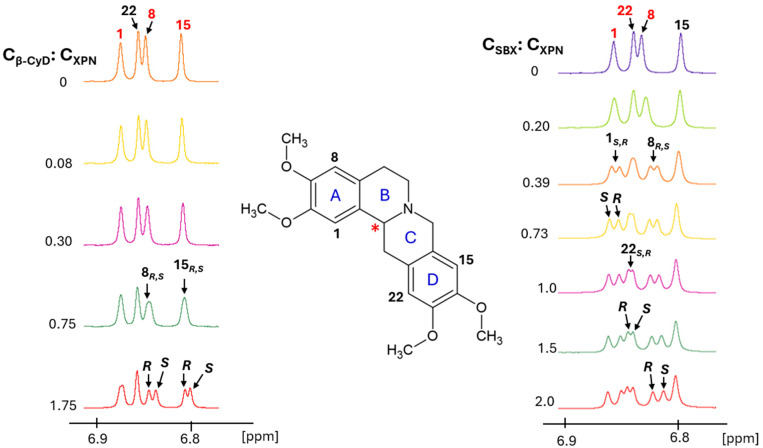
^1^H NMR spectra during β-CyD (**left**) and SBX (**right**) titration at varying selector-to-analyte ratios (pD 6.0). Chemical structure of XPN with the stereocenter marked by an asterisk, rings A–D labeled, and atom numbering (**in the middle**). Additional experimental conditions are described in Section 3.6.

Addition of SBX to racemic XPN resulted in chiral discrimination. At relatively low SBX amounts (selector-to-analyte ratio 0.39), splitting of the aromatic signals of the A-ring (H1 and H8) was already observed, and at 1:1 SBX-XPN molar ratio, the D-ring signal (H22) likewise displayed distinct splitting, indicating diastereomeric complex formation with interactions involving both aromatic rings. In contrast, with native β-CyD the aromatic signals first broadened at a β-CyD-to-XPN ratio of 0.75, while distinct splitting was observed only at a much higher host–guest ratio (1.75). This suggests a weaker stability of the β-CyD-XPN complexes and indicates a binding mode different from that with SBX. Additionally, a general upfield shift in aromatic protons was seen at approximately a 2:1 β-CyD-to-XPN ratio, supporting interaction with the CyD cavity (see Figure 4).

To assign the individual enantiomeric signals, pure (*S*)-XPN was added to the previously prepared samples containing racemic XPN and CyD (see Figure 4 and Supplementary Figures S10 and S11). Typically, (*S*)-XPN shifted upfield, while (*R*)-XPN shifted downfield, suggesting that the two enantiomers experience slightly different chemical environments in the presence of the chiral selector.

To provide direct evidence of inclusion complex formation, 2D ROESY NMR experiments were performed, as cross-peaks with the inner-cavity protons H3 and H5 of CyDs are indicative of host–guest spatial proximity.

In the case of β-CyD, extensive signal overlap in the 2D ROESY spectrum hindered reliable interpretation. The methoxy proton resonances of XPN overlapped with the H5 and H6 signals of β-CyD. To improve spectral separation, we attempted to adjust the pH and varied the β-CyD concentration during titration experiments; however, these proton resonances exhibited negligible chemical shift changes under all tested conditions. Consequently, any potential cross-peaks with H5 could not be unambiguously assigned, as they might result from intramolecular proximity between XPN’s methoxy and aromatic protons. To address this ambiguity, 1D ROESY experiments were also performed by selectively irradiating the aromatic signals of XPN. In this case, we expected to observe not only the trivial intramolecular methoxy correlations, but also potential intermolecular contacts with the H3 or H5 protons of β-CyD. However, these measurements remained inconclusive due to spectral overlap of the H5, H6, and methoxy signals. Additionally, no cross-peaks were observed with the inner-cavity H3 proton (see Supplementary Materials Figure S12). Although these limitations did not allow us to confirm inclusion with β-CyD, the absence of H3 correlations does not rule out complex formation, given the supporting evidence from NMR titration and successful enantioseparation observed in CE.

**Figure 5:**
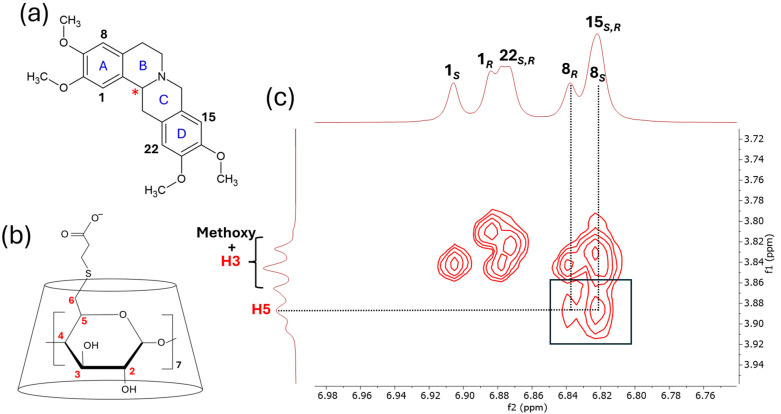
(**a**) XPN chemical structure with atom numbering, stereogenic center indicated by an asterisk and rings (A–D). (**b**) Schematic representation of SBX, (**c**) Partial 2D ROESY NMR spectra of the XPN–SBX (2.5:1) complex recorded in 30 mM pD 6.0 phosphate-buffered D_2_O, highlighting intermolecular cross-peaks between SBX H5 and XPN H8, H15 within the box. Additional experimental conditions are described in Section 3.6.

To overcome the spectral limitations, SBX—the selector exhibiting the highest enantioselectivity in CE—was selected for further NMR studies. In this case, methoxy signals of XPN and H5 of SBX do not overlap (see Figure S13 in the Supplementary Materials). The 2D ROESY spectra revealed clear cross-peaks between the aromatic H8 and H15 protons of XPN and the H5 proton of SBX (Figure 5), providing strong evidence for the simultaneous inclusion of both the A and D rings of XPN.

## 3. Materials and Methods

### 3.1. Materials

Racemic NOR was available from our previous study [28]. Melting points were determined in open capillary tubes without correction. Thin-layer chromatography was performed on aluminum sheets precoated with Kieselgel 60 F254 (Merck, Darmstadt, Germany), with a layer thickness of 0.2 mm. (*S*)-NOR was provided by Prof. Jean-Pierre Hurvois.

Chiralcel OD, Chiralpak AD, Chiralpak IA with identical dimensions (250 × 4.6 mm; particle size 10 µm) were obtained from Daicel (Tokyo, Japan). Astec Cyclobond I 2000 (250 × 4.6 mm; particle size 10 µm), were sourced from Sigma-Aldrich (St. Louis, MO, USA), while Nucleodex Beta-PM (200 × 4.0 mm; particle size 5 µm) and Chiral CD-Ph (250 × 4.6 mm; particle size 10 µm) chiral columns were purchased from Phenomenex (Torrance, CA, USA).

Native CyDs (α-, β-, and γ-CyD) as well as a wide range of chemically modified derivatives were also employed in this study. These included randomly methylated-γ-CyD (RAME-γ-CyD (DS ~12)), dimethylated-β-CyD (DIME-β-CyD, DS ~14), hydroxypropylated α-, β-, and γ-CyDs (HP-α-CyD, HP-β-CyD, HP-γ-CyD; each with DS ~3), and carboxymethylated derivatives (CM-α-CyD DS ~3.5, CM-β-CyD DS ~3, CM-γ-CyD DS ~4). Sulfated and sulfoalkylated derivatives such as sulfobutylether (SBE-α-, SBE-β-, SBE-γ-CyD), sulfopropylated (SP-β-, SP-γ-CyD), sulfated α-, β-, and γ-CyDs (S-α-CyD DS ~12, S-β-CyD DS ~13, S-γ-CyD DS ~14), single-isomer heptakis-(6-*O*-sulfo)-β-CyD (HS-β-CyD), heptakis-(2,3-*O*-dimethyl-6-*O*-sulfo)-β-CyD (HDMS), heptakis-(2,3-*O*-diacetyl-6-*O*-sulfo)-β-CyD (HDAS), heptakis-(2-*O*-methyl-3,6-*O*-disulfo)-β-CyD (HMDiSu), hexakis-(2,3-*O*-dimethyl-6-*O*-sulfo)-α-CyD (HxDMS), octakis-(2,3-*O*-dimethyl-6-*O*-sulfo)γ-CyD (ODMS), hexakis-(6-deoxy-6-(2-carboxyethyl)thio)-α-CyD (sualfadex, SAX), heptakis-(6-deoxy-6-(2-carboxyethyl)thio)-β-CyD (subetadex, SBX), octakis-(6-deoxy-6-(2-carboxyethyl)thio)-γ-CyD (sugammadex, SGX) were provided by CarboHyde Ltd. (Budapest, Hungary) and CycloLab Ltd. (Budapest, Hungary). 

Deuterium oxide (99.9% D) was obtained from Merck (Darmstadt, Germany). HPLC-grade MeOH and ACN were purchased from Merck, and DEA was sourced from Sigma-Aldrich (Budapest, Hungary). Additional reagents used for buffer preparation, rinsing, or as sample solvents—including sodium dihydrogen phosphate, acetic acid, tris(hydroxymethyl)aminomethane, sodium hydroxide, and dimethyl sulfoxide (DMSO)—were of analytical grade and purchased from commercial suppliers (Sigma-Aldrich, Budapest, Hungary). Bidistilled Millipore water (Burlington, MA, USA)was used throughout all experiments.

### 3.2. Synthesis of Racemic XPN

A mixture of NOR-HCl (5 g, 0.015 mol), 37% formaldehyde solution (20 mL), and 98% formic acid (40 mL) was heated in a water bath for 4 h. The pH of the reaction mixture was adjusted to 9–10 using a 10% sodium carbonate solution. The product was extracted with chloroform (3 × 30 mL), and the combined extracts were washed with brine and dried over sodium sulfate. Chloroform was evaporated in vacuo, and the residue was crystallized from ethanol. The crystals (3.1 g, 0.0087 mol, 58% yield) were filtered off, m.p.: 155–157 °C. literature m.p.: 158–159 °C [22]. The complete ^1^H and ^13^C NMR resonance assignments of XPN in MeOD along with the atomic numbering can be found in the Supplementary Materials Table S3, Figures S5, S14–S23.

### 3.3. Chiral HPLC

The analytical method followed our previously reported procedure [28]. Chiral HPLC analyses were performed on an Agilent 1100 system equipped with an inline degasser (G1322A), quaternary pump (G1311A), automatic injector (G1329A) with sample thermostat (G1330A), column thermostat (G1316A), and diode array detector (G1315A). The system was operated with Agilent ChemStation B04.03-SP2 software (Agilent Technologies, Bronnwald, Germany). Chromatographic screening was conducted at 25 °C in polar organic mode with a flow rate of 0.7 mL/min.

For semi-preparative separations, a Chiralpak AD column (250 × 10 mm, 10 µm) was used at 30 °C with MeOH:DEA (100:0.1, *v*/*v*) as the mobile phase. The injection volume was 20 µL, repeated over 100 injections. Sample was prepared at 20 mg/mL in methanol.

### 3.4. Circular Dichroism Spectroscopy

Circular dichroism spectra were recorded using a Jasco J-810 spectropolarimeter (Japan Spectroscopic Co., Tokyo, Japan). Enantiomer stock solutions were prepared in methanol at concentrations of 0.3 mg/mL for (*S*)-NOR and (*S*)-XPN 0.6 mg/mL for (*R*)-XPN and transferred to quartz cells with a 1 mm path length. Measurements were performed at 25 °C. Spectra were collected over the wavelength range of 220–325 nm. A total of five scans were accumulated for each sample. The spectrometer was operated in continuous scanning mode with a scanning speed of 50 nm/min, a bandwidth of 1 nm, and a data pitch of 0.2 nm. The response time was set to 4 s. Baseline correction and Savitzky–Golay smoothing were applied to the recorded spectra.

### 3.5. Capillary Electrophoresis

Capillary electrophoresis was carried out using an Agilent 7100 and HP 3D CE (Agilent Technologies, Waldbronn, Germany) equipped with a photodiode array detector and controlled via ChemStation software. Untreated fused silica capillaries (50 μm ID, 48.5 cm total length, 40 cm effective length) were obtained from Agilent. Initial conditioning of new capillaries was performed by sequential rinsing with 1 M NaOH, followed by 0.1 M NaOH, deionized water, and the appropriate buffer solution, each for 30 min. Prior to each analysis, the capillary was preconditioned by flushing with deionized water, the appropriate buffer, and the BGE, each for 2 min. Separations were performed at 25 °C with UV detection at 200 nm, applying a voltage of 15 kV. Samples were injected hydrodynamically at 150 mbar·s. The running buffers comprised 30 mM phosphate buffer at pH 7.4 (adjusted with 1 M NaOH) and 20 mM acetate buffer at pH 4.5 (adjusted with 1 M TRIS). CyDs were included in the BGE at concentrations between 0.25 and 30 mM. For screening, racemic XPN stock solution was prepared in methanol (1 mg/mL) and diluted with water as required.

To determine *K_stab_*, 0.1% DMSO was added as an EOF marker. Effective mobilities were calculated at varying CyD concentrations using CEval software (v. 0.6i3) [37]. Since affinity-based CE measurements frequently produce triangular peak shapes due to electromigration dispersion, the Haarhoff–Van der Linde (*HVL*) function (1) was applied to obtain more reliable estimates of the effective mobility:

(1) $${HVL}_{\delta }\left(t;a_{0},a_{1},a_{2},a_{3\delta }\right)=\frac{\frac{a_{0}}{a_{2}a_{3\delta }\sqrt{2\pi }}exp\left[{-\frac{1}{2}\left(\frac{t\;-a_{1}}{a_{2}}\right)}^{2}\right]}{\frac{1}{exp\left(a_{3\delta }\right)-1}+\frac{1}{2}\left[1+erf\left(\frac{t\;-a_{1}}{\sqrt{2a_{2}}}\right)\right]}$$

where *a*_0_ is the area of the *HVL* function, *a*_1_ the position of the Gaussian component (migration time of the analyte), *a*_2_ the standard deviation of the Gaussian component, and *a*_3*δ*_ triangular distortion.

Assuming a 1:1 analyte–CyD complexation and that the free CyD concentration approximates the total CyD concentration, the mobility–concentration relationship (2) follows:

(2) $$\mu_{eff}=\frac{\mu_{A}+{\;\mu }_{AS}K_{stab}\left[CyD\right]}{1+K_{stab}\left[CyD\right]}$$

where *μ_eff_* is the effective analyte mobility, *μ_A_* and *μ_AS_* are the mobilities of the free and complexed analyte, respectively, and *K_stab_* is the apparent complex stability constant.

Viscosity and ionic strength corrections were applied as described previously [38,39].

### 3.6. NMR Spectroscopy

NMR measurements were performed at 298 K using a Bruker Avance III spectrometer (Billerica, MA, USA) operating at 500 MHz for ^1^H and 125 MHz for ^13^C, and a Bruker Avance III spectrometer (Billerica, MA, USA) operating at 400 MHz for ^1^H and 100 MHz for ^13^C. Standard pulse sequences from the TopSpin software (v. 3.6.2.) library were employed for all experiments. Conventional two-dimensional NMR experiments, including ^1^H–^1^H COSY, ^1^H–^1^H ROESY, ^1^H–^13^C HSQC, and ^1^H–^13^C HMBC, were recorded in MeOD for structure elucidation after synthesis and in D_2_O containing 30 mM phosphate buffer (pD 6.0) with 2 mM racemic XPN. For ^1^H NMR titration experiments, 10 mM solutions of β-CyD and SBX were prepared in the same buffer system. For spiking experiments, a 2 mM stock solution of (*S*)-XPN was prepared in 30 mM phosphate buffer (pD 6.0) and added to the racemic sample. One-dimensional and two-dimensional ROESY spectra were acquired with a mixing time of 300 ms.

## 4. Conclusions

This study presents a comprehensive strategy for the enantiomeric separation of XPN, a pharmacologically relevant THPB alkaloid. As a result of CE screening, anionic, β-cavity-sized selectors consistently provided superior enantioseparation performance, likely due to favorable electrostatic interactions with the positively charged XPN. Notably, the incorporation of 2-carboxyethylthio side chains in SBX markedly enhanced enantioselectivity, emphasizing the role of β-CD derivatization in chiral recognition.

Semi-preparative HPLC enabled the isolation of XPN enantiomers and thus the determination of the EMO. The migration order remained consistent across most selectors, except for SBE-β-CyD, where an inversion occurred, possibly due to secondary interactions with its sulfobutyl side chains.

^1^H NMR titration studies revealed enantiodifferentiation involving both the A and D rings of XPN, indicating multi-site interaction. This diastereomeric splitting pattern aligns with previous observations on SGX-LAU complexes, suggesting related recognition modes. The observed differences in binding behavior appear to stem from the structural properties of the alkaloids: XPN’s rigidity favors deeper inclusion in SBX, while the more flexible LAU interacts primarily through its D-ring with the larger SGX cavity.

By establishing a robust workflow combining separation and structural elucidation, this study provides a strong basis for the chiral analysis of THPBs, supporting future applications in quality control, pharmacological research, and natural product studies.

## Figures and Tables

**Table 1:** Retention times of the first enantiomer (t_1_), resolution values (*R_s_*) of XPN applying six different analytical chiral columns, with MeOH or ACN as mobile phase with 0.1% diethylamine. Further conditions can be found in Section 3.3.

Column	Mobile Phase	t_1_	*R_s_*
Chiral CyD-Ph	ACN	5.97	-
	MeOH	11.42	1.9
Cyclobond I	ACN	6.11	-
	MeOH	4.93	-
Nucleodex β-CyD	ACN	2.70	-
	MeOH	2.97	-
Chiralcel OD	ACN	6.58	0.60
	MeOH	9.10	0.60
Chiralpak AD	ACN	5.74	-
	MeOH	7.46	5.50
Chiralpak IA	ACN	3.84	-
	MeOH	5.64	2.90

**Table 2:** The maximum resolution values (*R_s_*) and enantiomer migration order (EMO) of XPN were determined using different CyDs in a 20 mM acetate-TRIS buffer at pH 4.5 and a 30 mM phosphate buffer at pH 7.4, at a temperature of 25 °C, with an applied voltage of 15 kV and a wavelength of 200 nm. Further conditions can be found in Section 3.5. For the abbreviation of the CyDs, see Section 2.3 and Section 3.1.

CyDs	Concentration	pH 4.5		pH 7.4	
	(mM)	*R_s_*	EMO	*R_s_*	EMO
β-CyD	10	-	-	1.4	*R*,*S*
S-β-CyD	10	3.2	*R*,*S*	<1	*R*,*S*
SBE-β-CyD	10	4.6	*S*,*R*	<1	*S*,*R*
CM-β-CyD	10	-	-	<0.5	-
SBX	2.5	n.d.	n.d.	9.3	*R*,*S*
SGX	7.5	n.d.	n.d.	1.4	*R*,*S*

n.d. not determined.

## Data Availability

Data are contained within the article and Supplementary Materials.

## Supplementary Materials

The following supporting information can be downloaded at https://www.mdpi.com/article/10.3390/ijms26199405/s1.
