# Supplementary material for: Enantioselective Complexation of Xylopinine: A Cyclodextrin-Assisted CE and NMR Study
Source: Int J Mol Sci. 2025 Sep 26;26(19):9405. doi: 10.3390/ijms26199405 (PMC12524373; doi:10.3390/ijms26199405)
Supplement: Supplementary file 1 [file ijms-26-09405-s001.zip › ijms-3876531-supplementary.pdf]

Supplementary Material for Article

Enantioselective Complexation of Xylopinine: A Cyclodextrin-Assisted CE and NMR study

Erzsébet Várnagy [1,2], Gergő Tóth [2,3], Sándor Hosztafi [2,3], Milo Malanga [4], Ida Fejős [1,2] and Szabolcs Béni [5,*]

[1] Department of Pharmacognosy, Semmelweis University, Üllői út 26, H-1085 Budapest, Hungary; varnagy.erzsebet@phd.semmelweis.hu; fejos.ida@semmelweis.hu
[2] Center for Pharmacology and Drug Research & Development, Semmelweis University, Budapest, Hungary
[3] Department of Pharmaceutical Chemistry, Semmelweis University, Hőgyes Endre u. 9, H-1092 Budapest, Hungary; toth.gergo@semmelweis.hu; hosztafi.sandor@semmelweis.hu
[4] CarboHyde Ltd, Budapest; milo.malanga@carbohyde.com
[5] Integrative Health and Environmental Analysis Research Laboratory, Department of Analytical Chemistry, Institute of Chemistry, ELTE Eötvös Loránd University, Pázmány Péter sétány 1/A, H-1117 Budapest, Hungary; szabolcs.beni@ttk.elte.hu
* Correspondence: szabolcs.beni@ttk.elte.hu

Contents

Circular Dichroism measurements 2
CE measurements 3
NMR measurements 5
Following the synthetic procedures by NMR 10

Circular Dichroism measurements

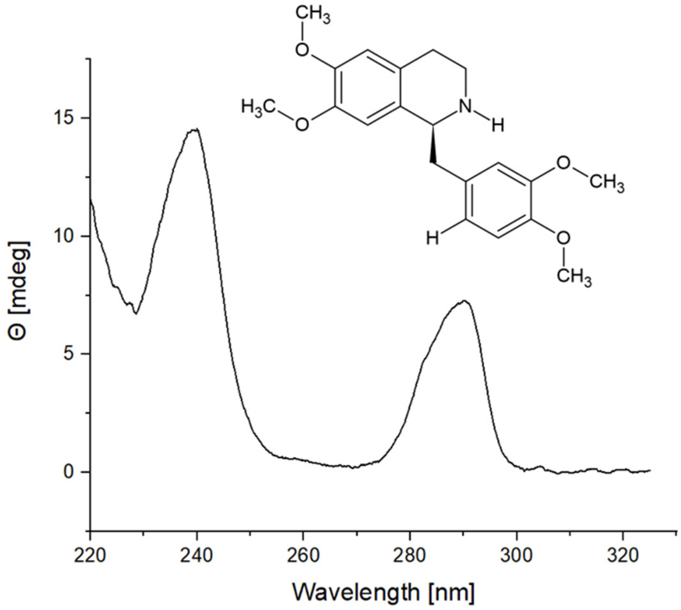

Figure S1. Circular dichroism spectra of (*S*)-norlaudanosine ((*S*)-NOR). (0.3 mg/mL in MeOH). Further conditions can be found in Section 3.4.

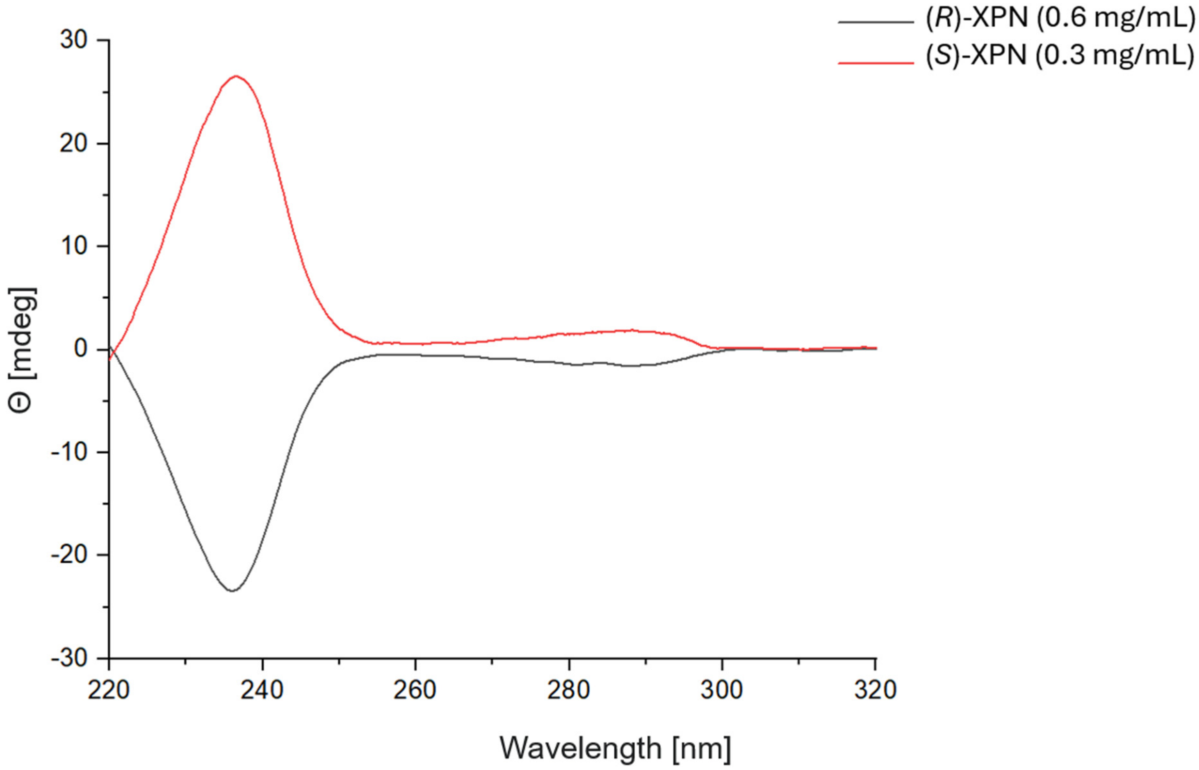

Figure S2. Circular dichroism spectra of (*R*)- and (*S*)-xylopinine ((*S*)-XPN). Further conditions can be found in Section 3.4.

CE measurements

Table S1. Averaged apparent XPN–cyclodextrin (CyD) complex stability constants (K_{stab}, / M^{-1}) and complex mobilities ($\mu_{(AS)}$ / 10^{-5} $cm^2V^{-1}s^{-1}$) measured by affinity capillary electrophoresis at 30 mM phosphate buffer (pH 7.4), 25 °C, 15 kV, 200 nm. Further conditions can be found in Section 3.5.

CyDs		(*R*)-XPN	(*S*)-XPN
β-CyD	K_{stab}	280 ± 110	380 ± 70
	$\mu_{(AS)}$	0.9 ± 0.3	0.3 ± 0.1
Subetadex	K_{stab}	2105 ± 70	3830 ± 180
	$\mu_{(AS)}$	-33.96 ± 0.5	-33.95 ± 0.5

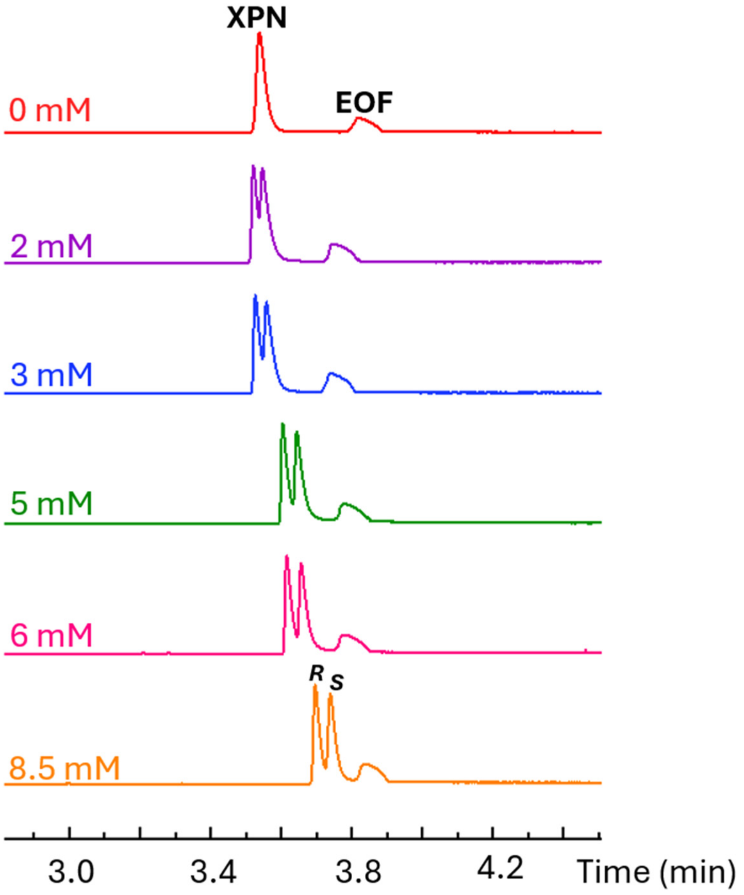

Figure S3. Representative electropherograms obtained using various β-CyD concentrations, showing the analyte, XPN and the electroosmotic flow (EOF). Additional experimental conditions are provided in Section 3.5.

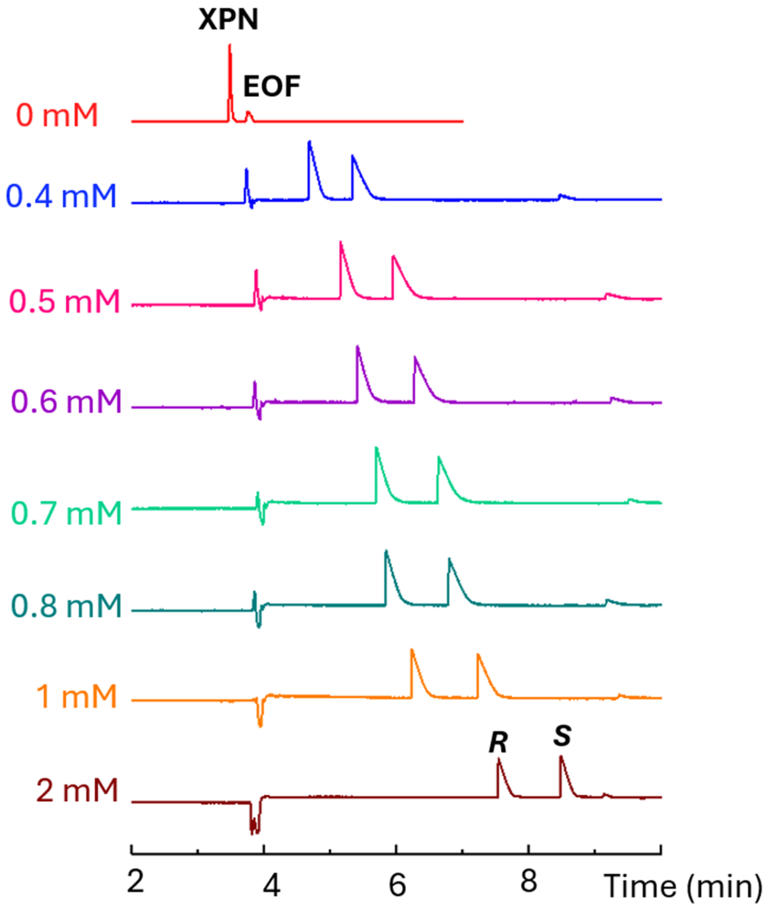

Figure S4. Representative electropherograms obtained using subetadex (SBX) at various concentrations, showing the analyte, XPN and the electroosmotic flow (EOF). Additional experimental conditions are provided in Section 3.5.

NMR measurements

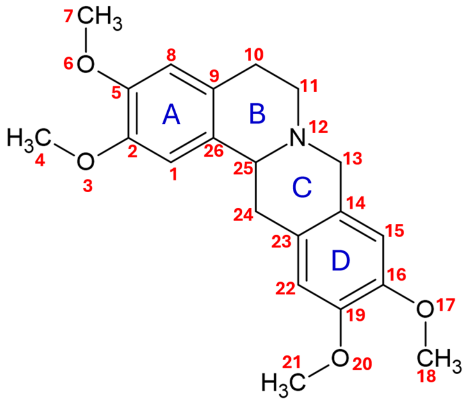

Figure S5. The structure of XPN with atom numbering and ring labels (A-D).

Table S2. ^{1}H assignment of XPN, (in D_2O; pD 6.0; 500 MHz). For the atomic positions of XPN see Figure S5.

Atomic position	[1]H δ (ppm)
1	6.90 (s, 1H)
2	-
3	-
4	3.81 (s, 3H)
5	-
6	-
7	3.79 (s, 3H)
8	6.86 (s, 1H)
9	-
10a	2.97-3.07 (m, broad, 1H)
10b	3.14-3.23 (m, broad, 1H)
11a	3.45 (dt, J= 4.9; 11.5 Hz, 1H)
11b	3.73 (m, broad, 1H)
12	-
13a	4.45 (m, 1H)
13b	4.45 (m, 1H)
14	-
15	6.82 (s, 1H)
16	-
17	-
18	3.77 (s, 3H)
19	-
20	-
21	3.79 (s, 3H)
22	6.87 (s, 1H)
23	-
24a	2.97-3.07 (dd, J= 17.0, 1H)
24b	3.64 (m, broad, 1H)
25	4.62 (overlapped by solvent, 1H)
26	-

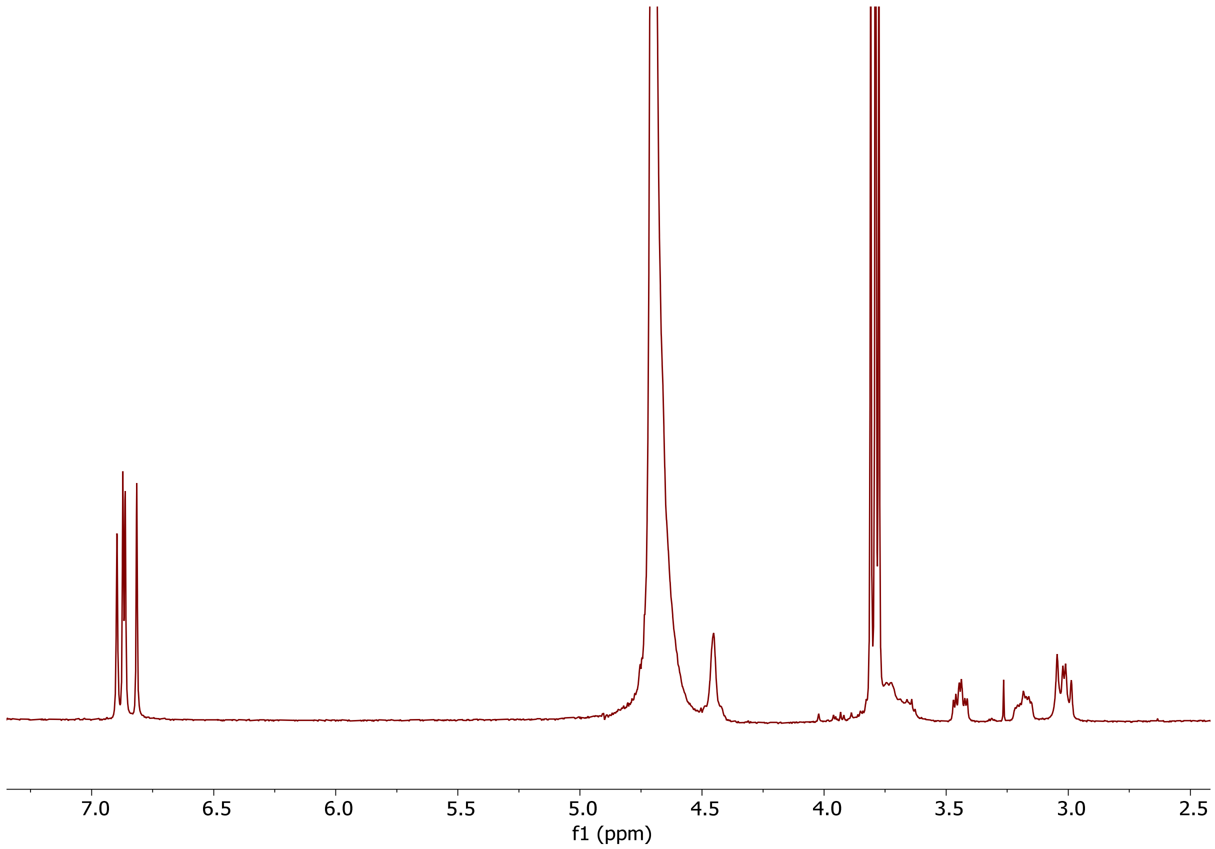

Figure S6. ^{1}H NMR spectrum of racemic XPN (in D_2O; pD 6.0; 500 MHz).

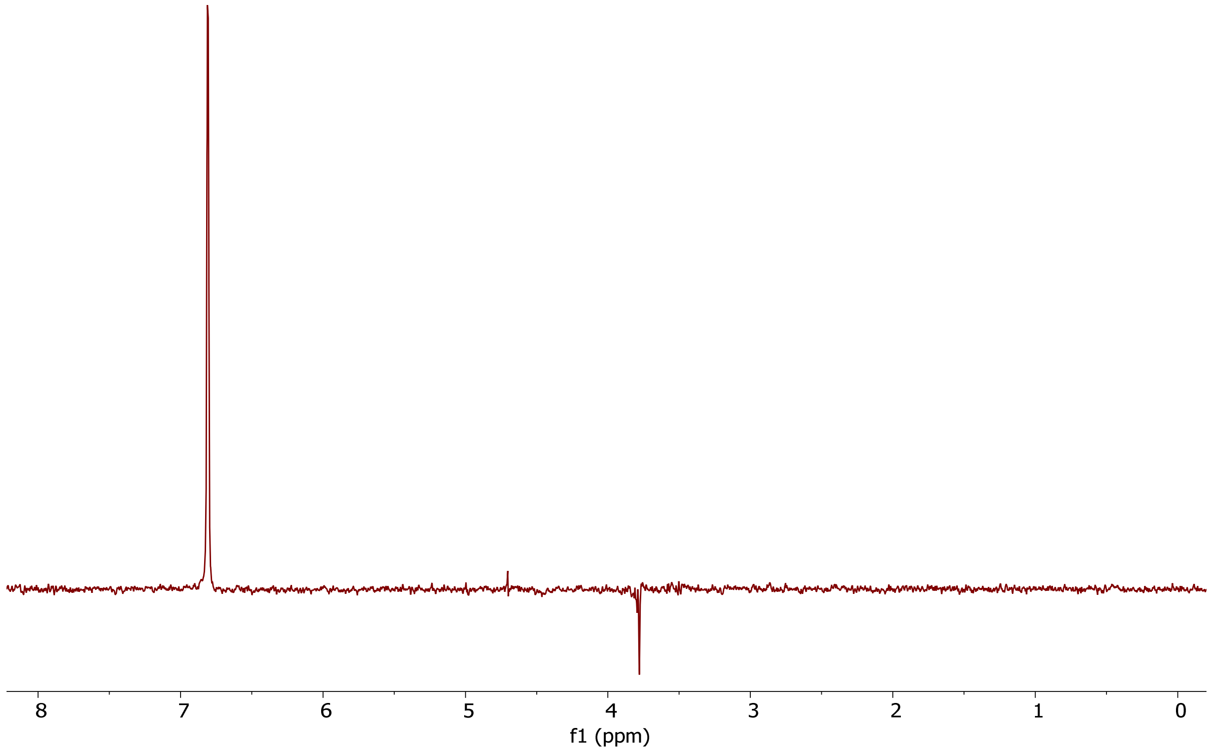

Figure S7. 1D selective ROESY spectrum of racemic XPN, the identification of H18 methoxy signal by irradiation (in D_2O; pD 6.0; 500 MHz).

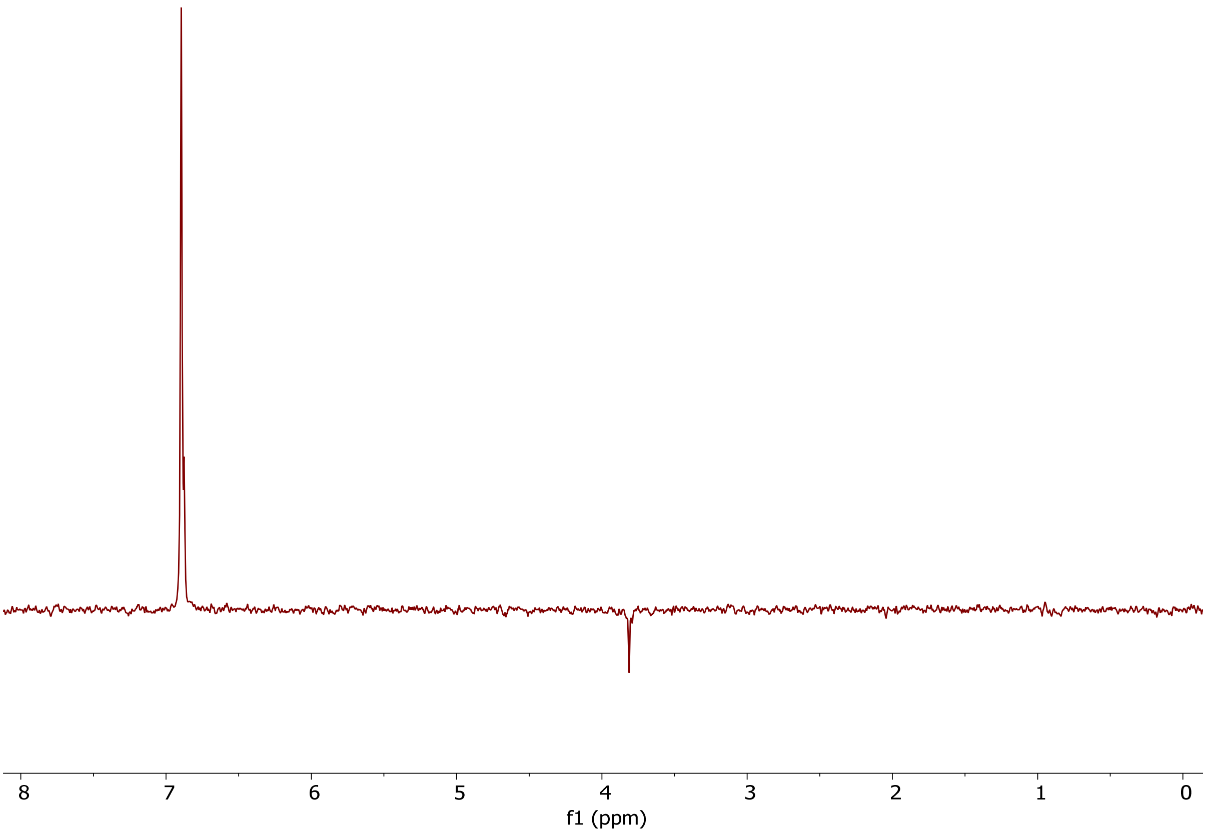

Figure S8. 1D selective ROESY spectrum of racemic XPN, the identification of H7 methoxy signal by irradiation (in D_2O; pD 6.0; 500 MHz).

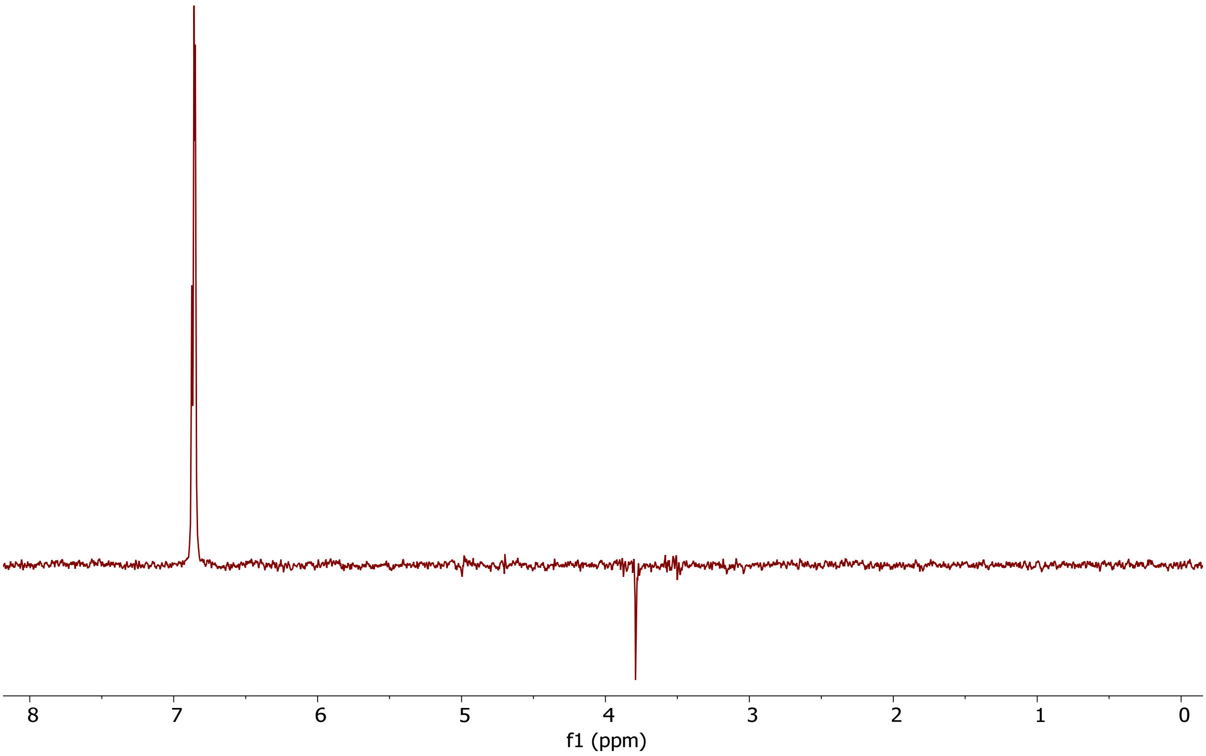

Figure S9. 1D selective ROESY spectrum of racemic XPN, the identification of H4 and H21 methoxy signals by irradiation (in D_2O; pD 6.0; 500 MHz).

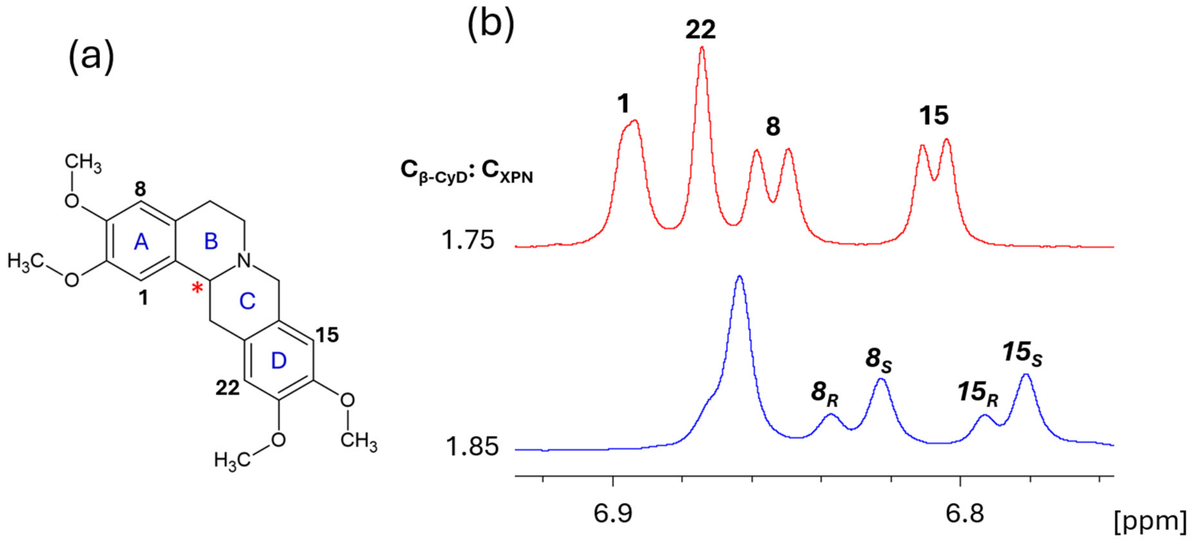

Figure S10. (a) The structure of XPN, labelled its aromatic protons, stereogenic center indicated by an asterisk and rings (A–D). (b) Partial ^{1}H spectra of the β-CyD-XPN complex (1.75:1) and (1.85:1) spiked with (*S*)-XPN. Further conditions can be found in Section 3.6.

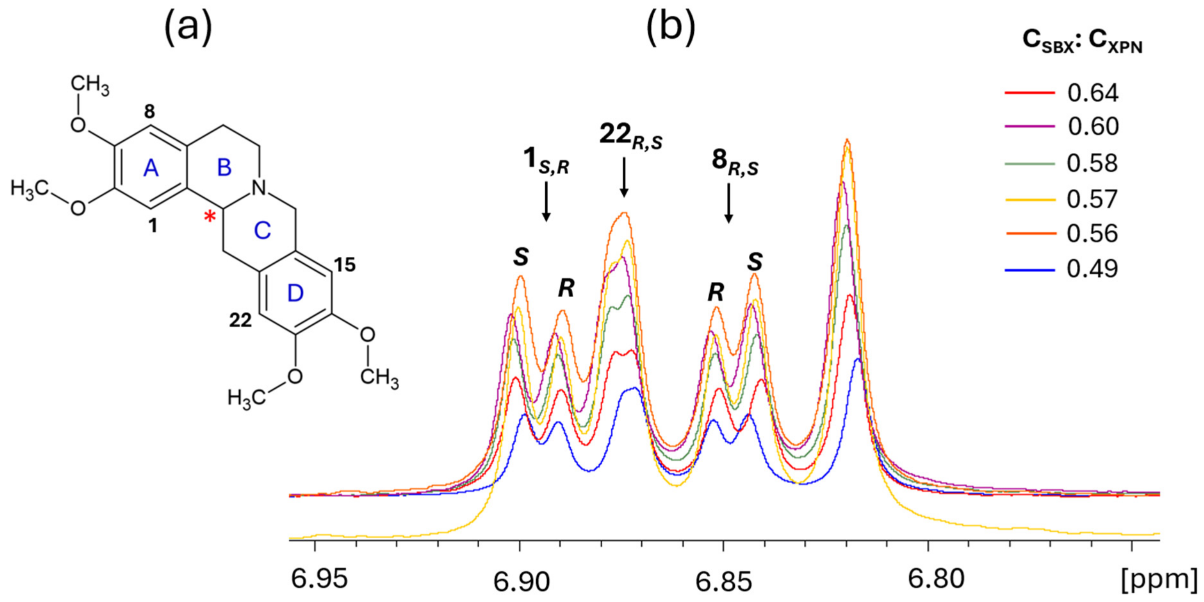

Figure S11. (a) The structure of XPN, labelled its aromatic protons. (b) Partial ^{1}H spectra of the SBX-XPN complex spiked with (*S*)-XPN. Further conditions can be found in Section 3.6.

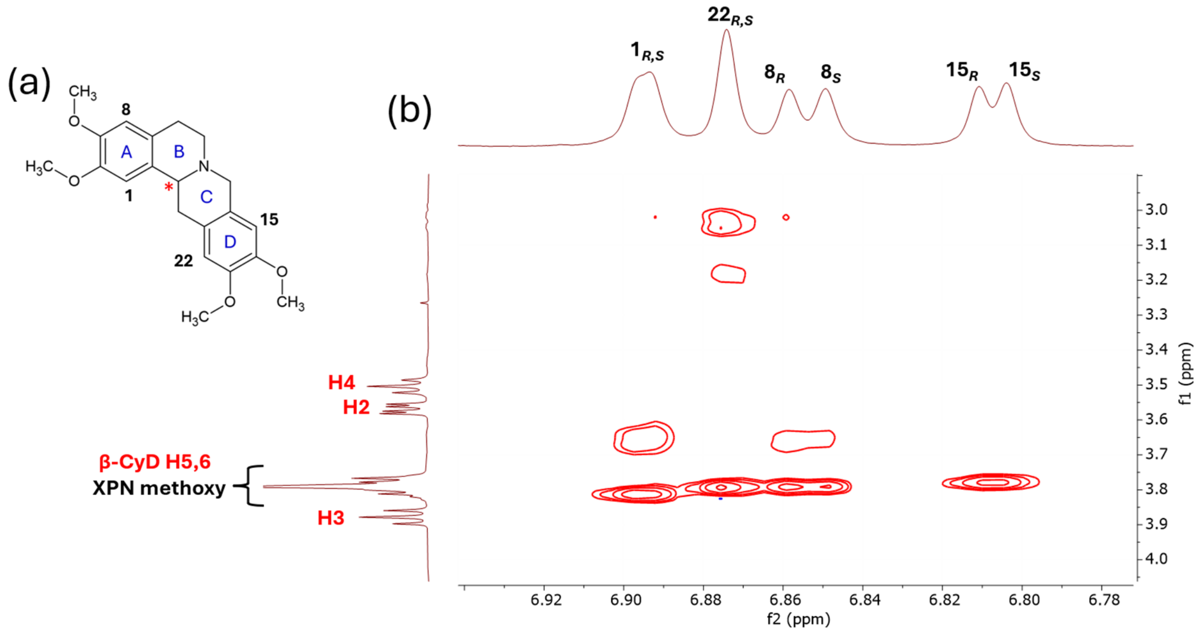

Figure S12. (a) The structure of XPN, labelled its aromatic protons. (b) Partial 2D ROESY spectra of the β-CyD-XPN complex (1.75:1). Further conditions can be found in Section 3.6.

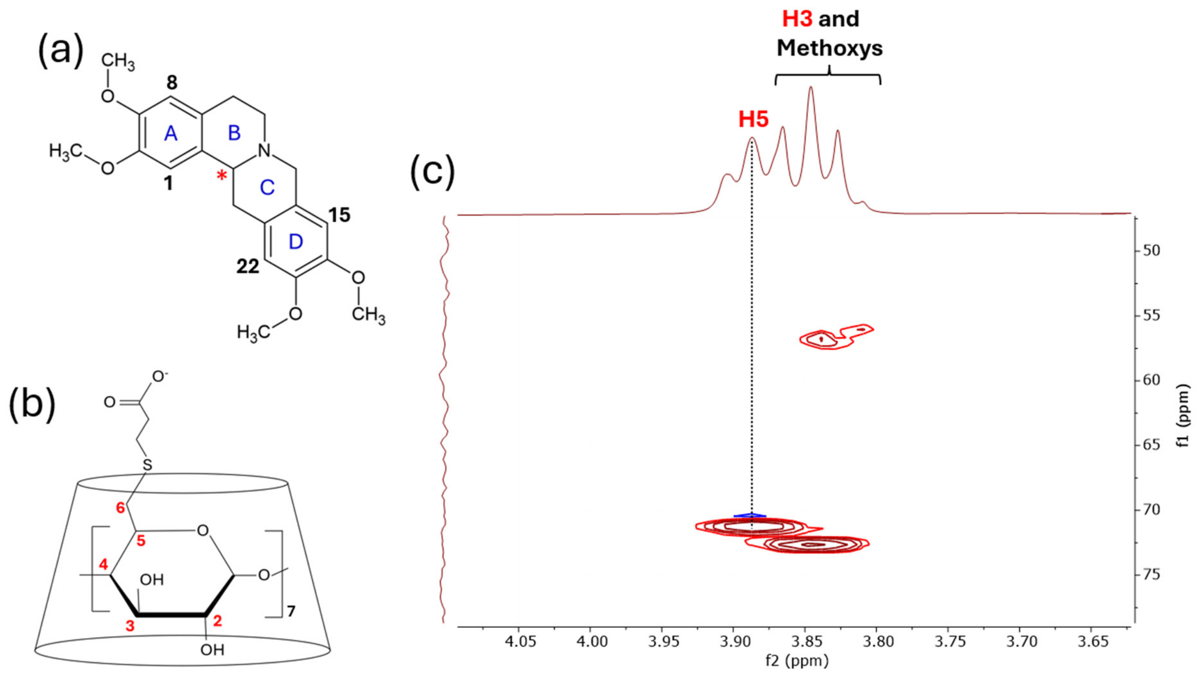

Figure S13. (a) The structure of XPN, labelled its aromatic protons. (b) Schematic representation of SBX, (c) Partial HSQC spectra of SBX-XPN complex (2.5:1). Further conditions can be found in Section 3.6.

Following the synthetic procedures by NMR

Table S3. ^{1}H and ^{13}C assignment of XPN, (in MeOD, 500 MHz). For the atomic positions of XPN see Figure S5.

**Atomic position**	**^{1}H δ (ppm)**	**^{13}C δ (ppm)**
1	6.90 (s, 1H)	109.0
2	-	147.7
3	-	-
4	3.86 (s, 3H)	55.3
5	-	147.9
6	-	-
7	3.83 (s, 3H)	55.1
8	6.73 (s, 1H)	111.6
9	-	126.4
10a	2.73 (m, broad, 1H)	28.0
10b	3.11 (m, broad, 1H)	28.0
11a	2.64 (dt, J=3.9; 11.5 Hz, 1H)	51.2
11b	3.20 (ddd, J=1.8; 5.6; 7.4 Hz, 1H)	51.2
12	-	-
13a	3.67 (d, J=14.8 Hz, 1H)	57.6
13b	3.98 (d, J=14.8 Hz, 1H)	57.6
14	-	126.2
15	6.73 (s, 1H)	109.3
16	-	147.7
17	-	-
18	3.82 (s, 3H)	55.1
19	-	148.0
20	-	-
21	3.83 (s, 3H)	55.1
22	6.82 (s, 1H)	111.7
23	-	125.8
24a	2.79 (dd J= 16.0; 11.5 Hz, 1H)	35.3
24b	3.45 (dd, J= 11.3; 4.2, 1H)	35.3
25	3.62 (dd, J=11.3; 3.9 Hz, 1H)	59.7
26	-	129.4

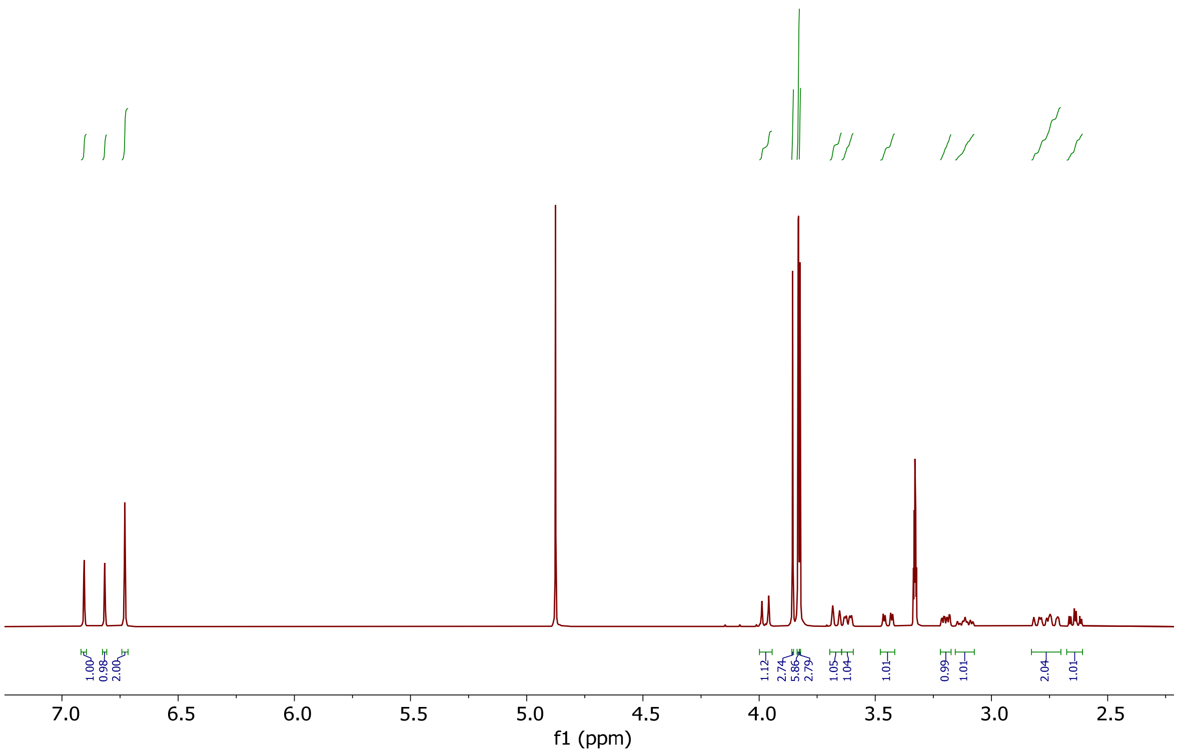

Figure S14. ^{1}H NMR spectrum of racemic XPN (in MeOD, 298 K, 500 MHz).

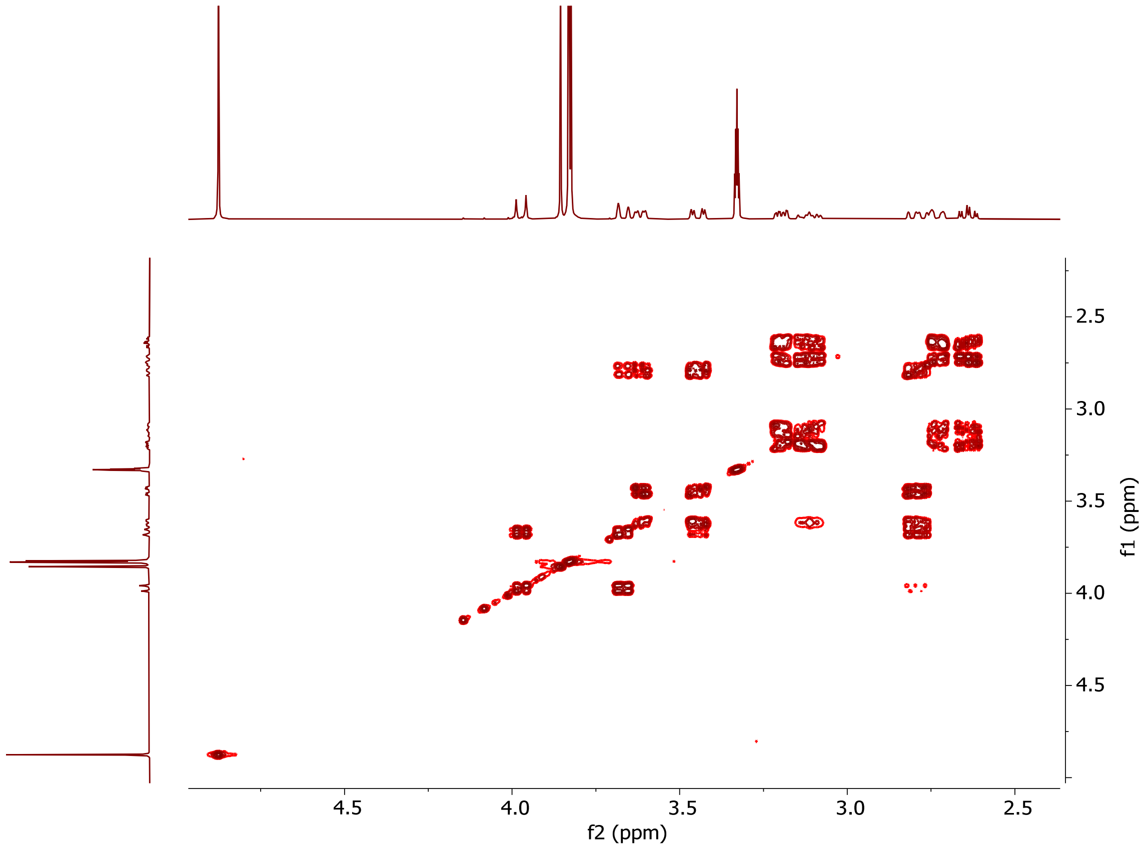

Figure S15. COSY spectrum of racemic XPN, aliphatic region (in MeOD, 298 K, 500 MHz).

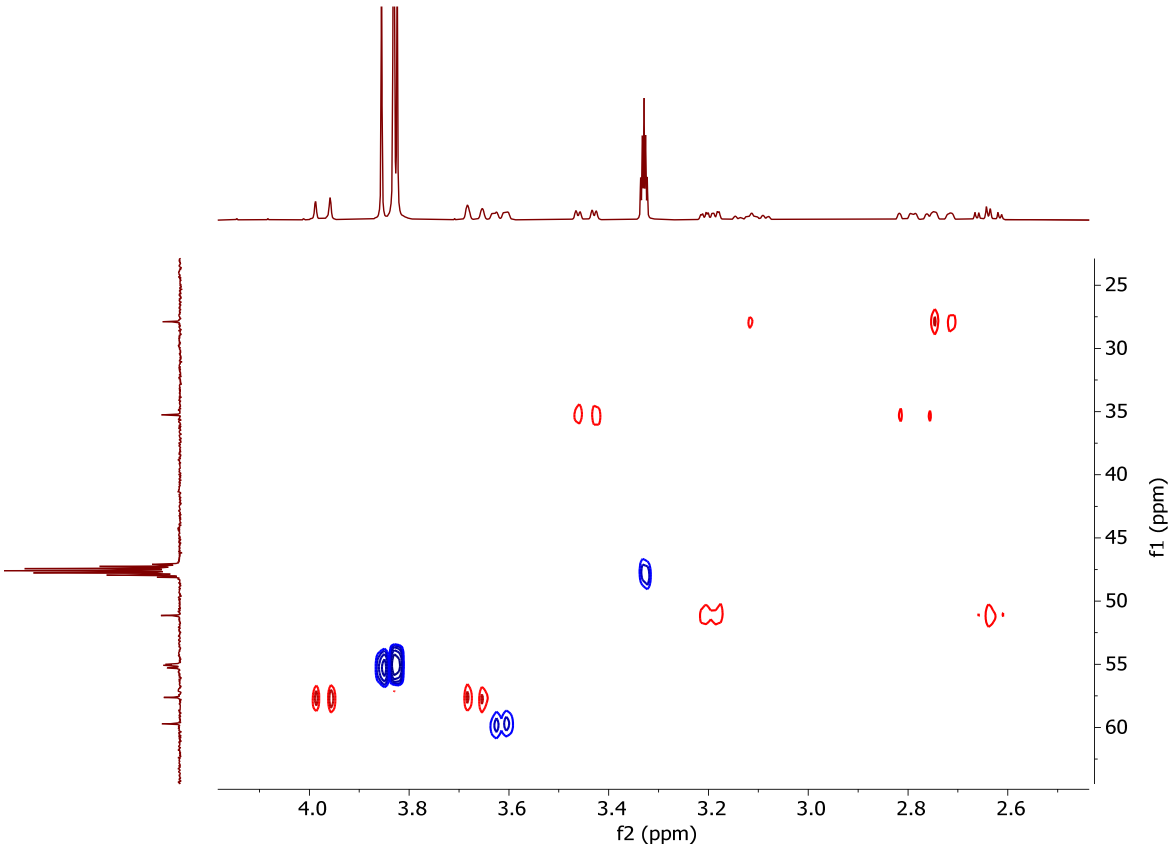

Figure S16. DEPT-edited HSQC spectrum of racemic XPN, aliphatic region (in MeOD, 298 K, 500 MHz).

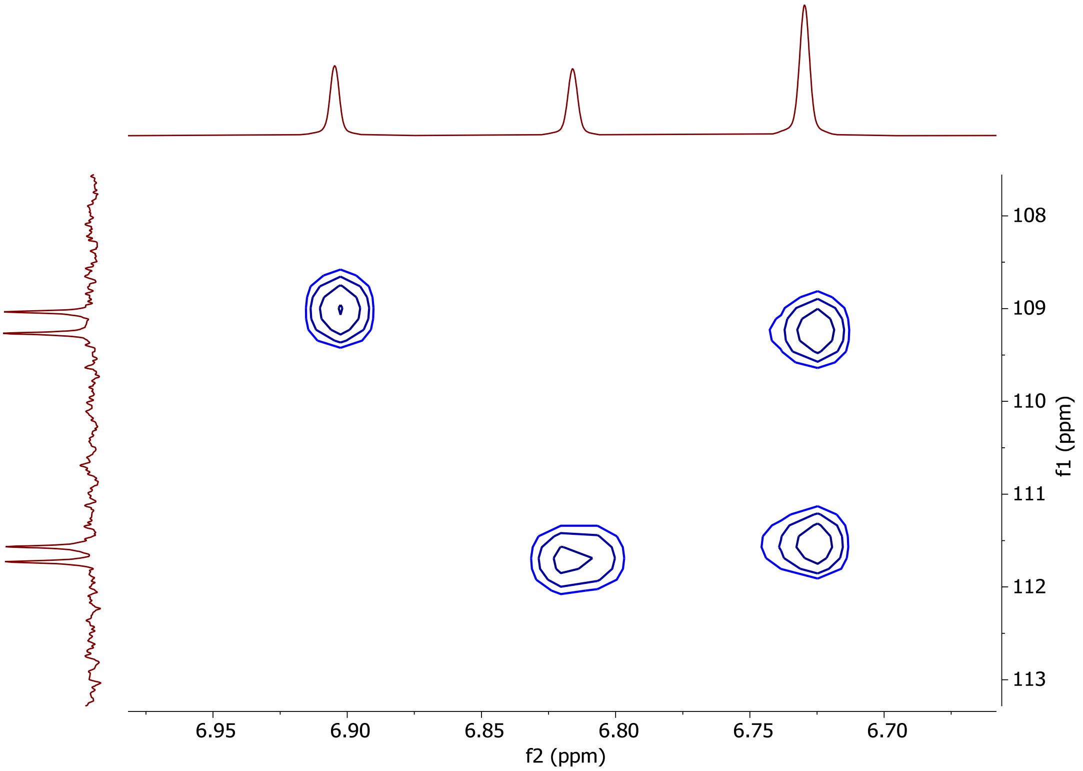

Figure S17. DEPT-edited HSQC spectrum of racemic XPN, aromatic region (in MeOD, 298 K, 500 MHz).

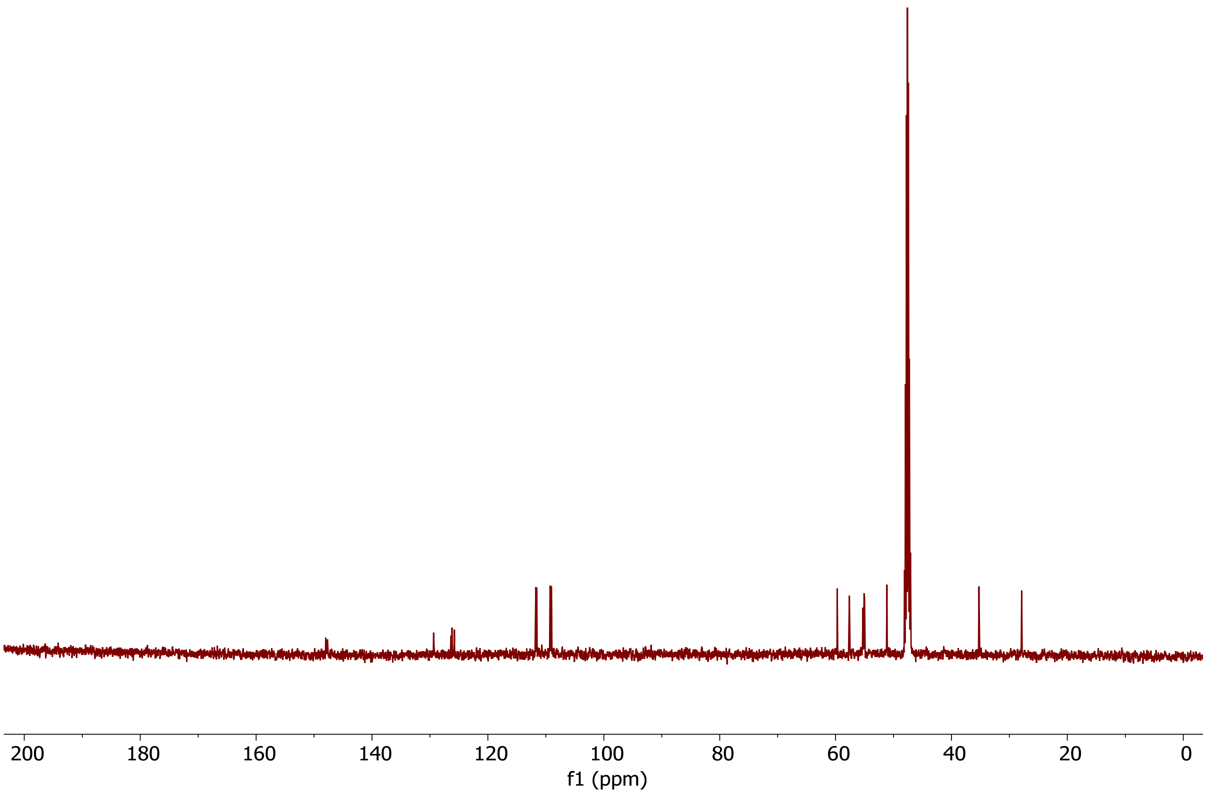

Figure S18. ^{13}C spectrum of racemic XPN (in MeOD, 298 K, 500 MHz).

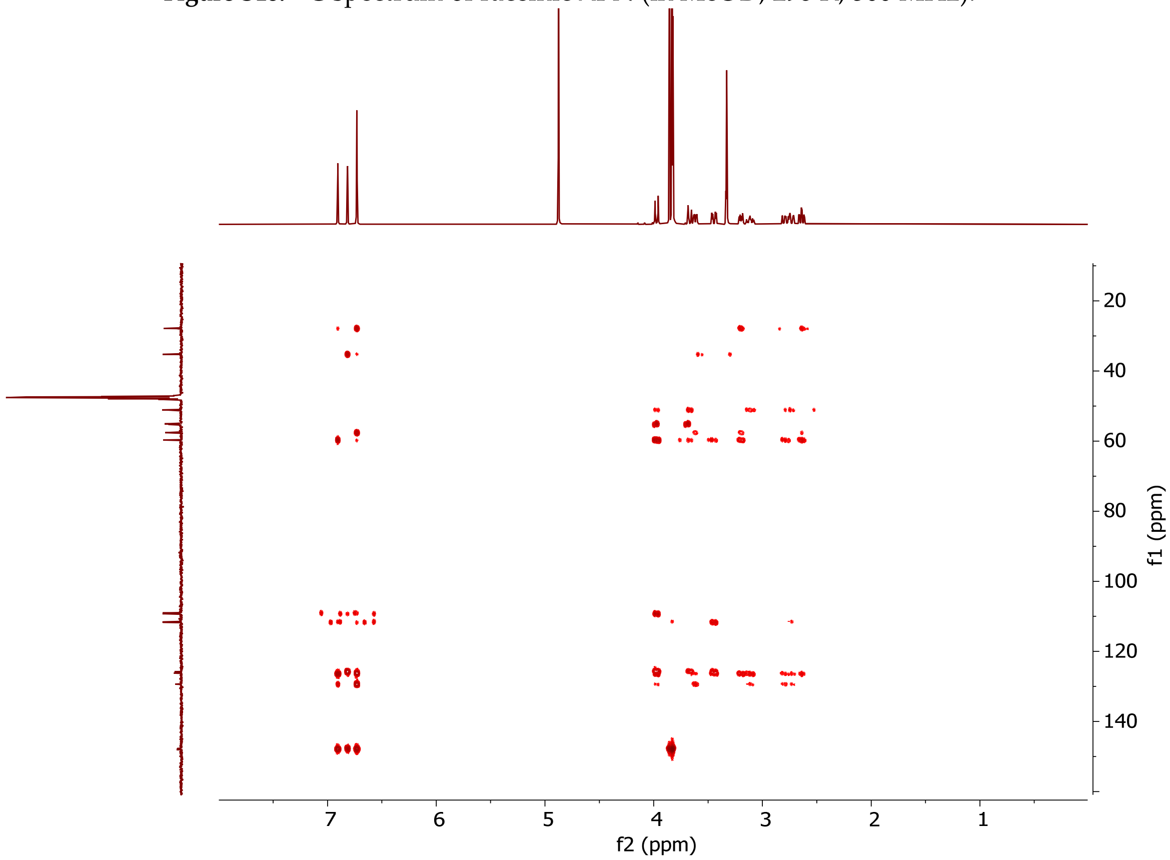

Figure S19. HMBC spectrum of racemic XPN (in MeOD, 298 K, 500 MHz).

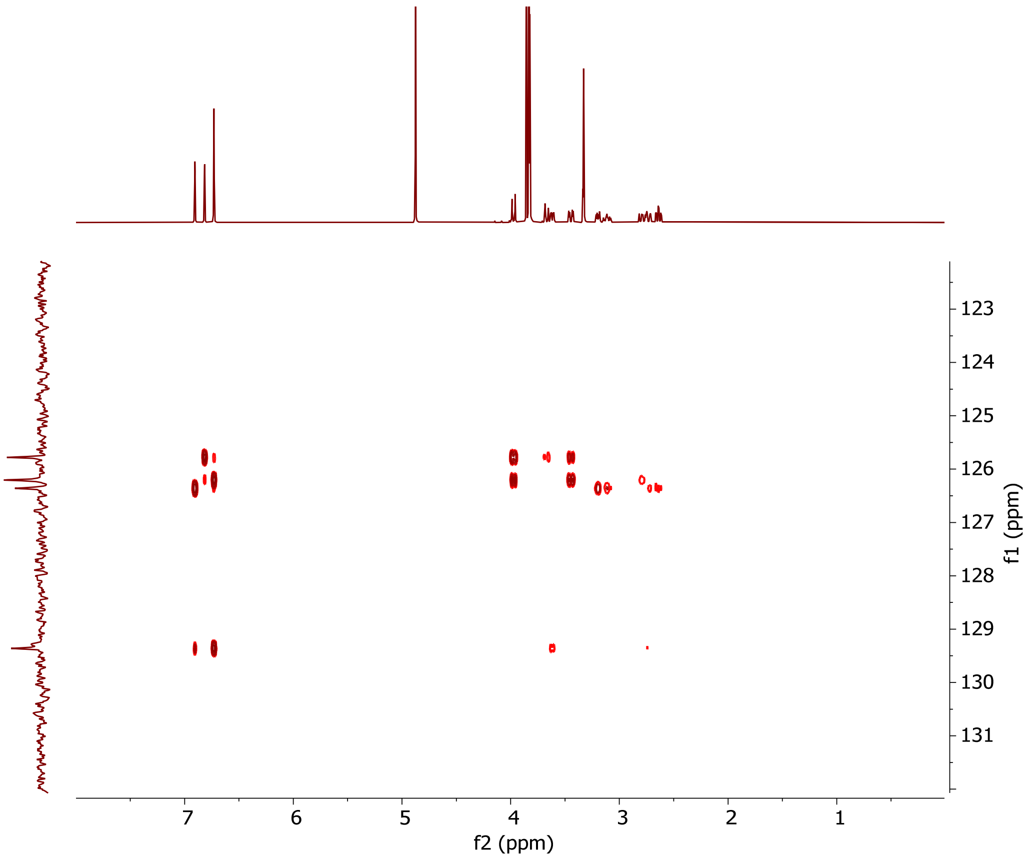

Figure S20. 2D selective HMBC spectrum of racemic XPN (in MeOD, 298 K, 500 MHz).

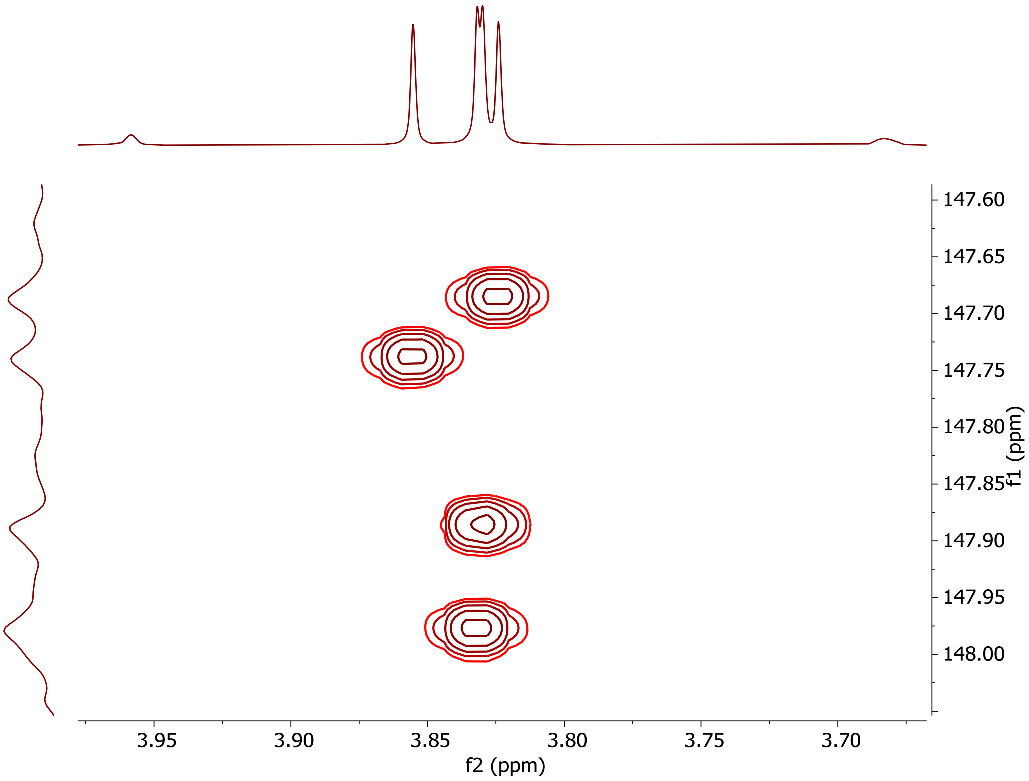

Figure S21. 2D selective HMBC spectrum of racemic XPN (in MeOD, 298 K, 500 MHz).

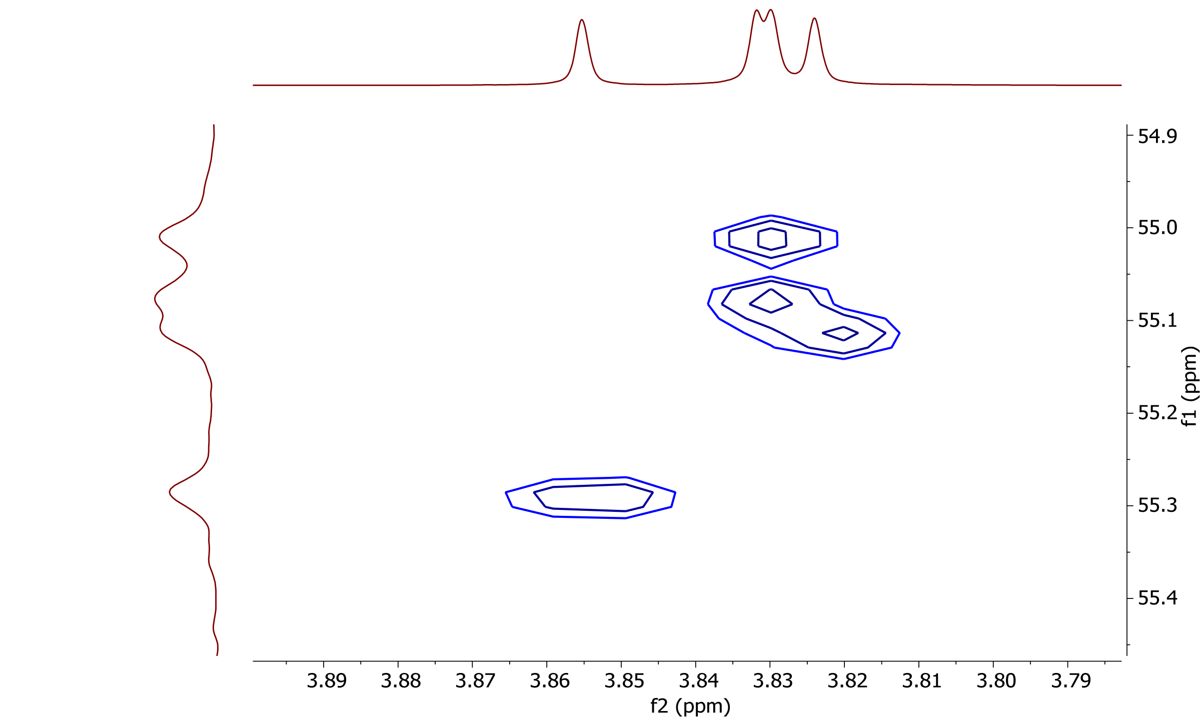

Figure S22. 2D selective HMBC spectrum of racemic XPN (in MeOD, 298 K, 500 MHz).

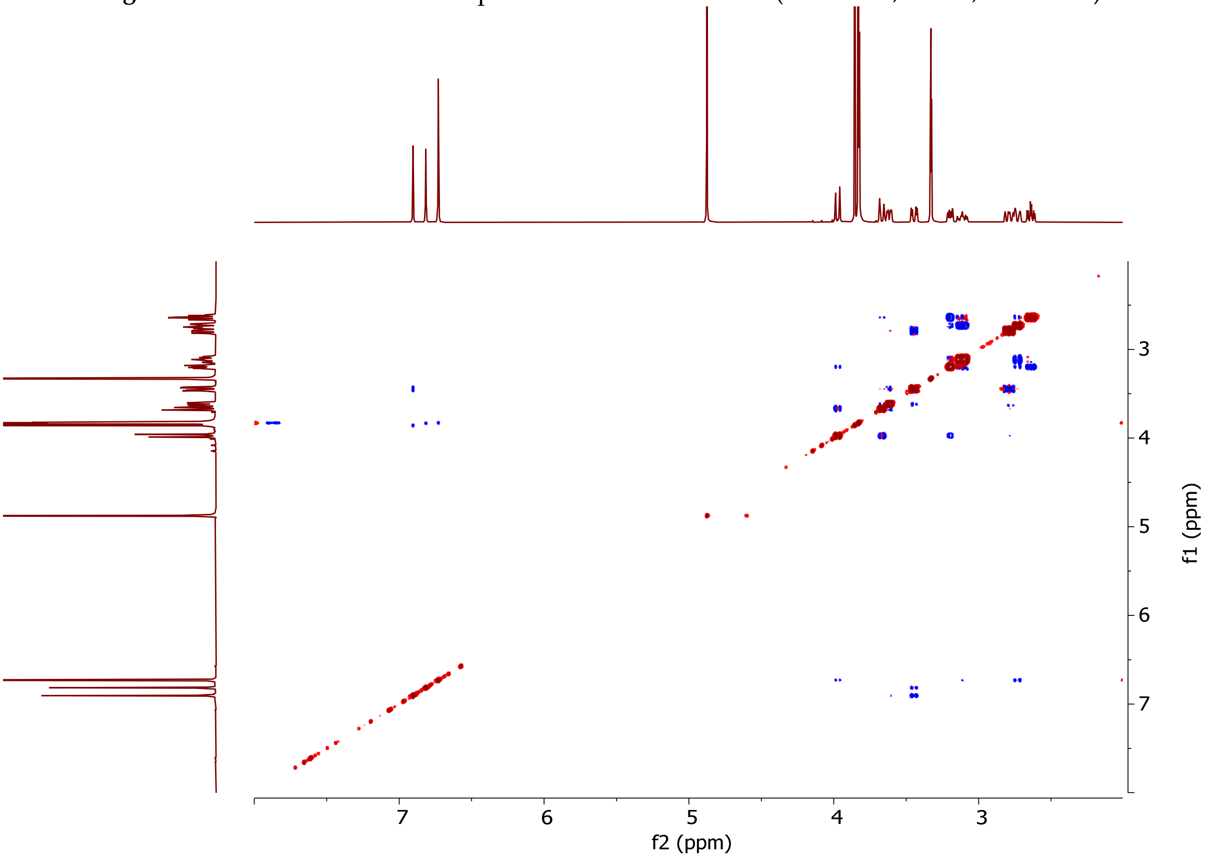

Figure S23. NOESY spectrum of racemic XPN (in MeOD, 298 K, 500 MHz).
